# Recent advances in managing HIV-associated cryptococcal meningitis

**DOI:** 10.12688/f1000research.17673.1

**Published:** 2019-05-28

**Authors:** Timothée Boyer-Chammard, Elvis Temfack, Alexandre Alanio, Joseph N. Jarvis, Thomas S. Harrison, Olivier Lortholary

**Affiliations:** 1 Molecular Mycology Unit and National Reference Centre for Invasive Mycoses, UMR2000, CNRS, Institut Pasteur, Paris, France; 2 Université de Paris, Necker Pasteur Center for Infectious Diseases and Tropical Medicine, Hôpital Necker Enfants malades, AP-HP, IHU Imagine, Paris, France; 3 Douala General Hospital, Douala, Cameroon; 4 University Paris Diderot, Sorbonne Paris Cité, Université de Paris, Laboratory of Parasitology-Mycology, Saint-Louis Hospital, APHP, Paris, France; 5 Department of Clinical Research, Faculty of Infectious and Tropical Diseases, London School of Hygiene and Tropical Medicine, London, UK; 6 Botswana Harvard AIDS Institute Partnership, Gaborone, Botswana; 7 Institute of Infection and Immunity, St. George’s University of London, London, UK

**Keywords:** Cryptococcus neoformans, cryptococcal meningo-encephalitis, Amphotericin B, Ambisome, Fluconazole, Flucytosine, advanced HIV disease, AIDS

## Abstract

The recent development of highly sensitive and specific point-of-care tests has made it possible to diagnose HIV-associated cryptococcal meningitis within minutes. However, diagnostic advances have not been matched by new antifungal drugs and treatment still relies on old off-patent drugs: amphotericin B, flucytosine and fluconazole. Cryptococcal meningitis treatment is divided in three phases: induction, consolidation and maintenance. The induction phase, aimed at drastically reducing cerebrospinal fluid fungal burden, is key for patient survival. The major challenge in cryptococcal meningitis management has been the optimisation of induction phase treatment using the limited number of available medications, and major progress has recently been made. In this review, we summarise data from key trials which form the basis of current treatment recommendations for HIV-associated cryptococcal meningitis.

## Introduction

Cryptococcal meningitis (CM), a severe infection that is fatal without treatment, occurs primarily in patients with impaired cell-mediated immunity ^1^. Prior to the human immunodeficiency virus (HIV) pandemic, most cases were described in solid-organ-transplant patients ^2^. However, since 1993, CM became an acquired immune deficiency syndrome (AIDS)-defining infection in HIV-infected patients, and the number of cases increased rapidly, especially in Sub-Saharan Africa (SSA), where more than 90% of cases are HIV-associated ^3^. In 2014, it was estimated that 223,000 cases of CM occurred globally, causing over 180,000 deaths, three quarters of which occurred in SSA, making CM the leading cause of adult meningitis in the region, responsible for 15% of all HIV-associated mortality ^4^.

Ideally, prevention of HIV-related CM relies on prevention of severe immune suppression through early HIV diagnosis and initiation of antiretroviral therapy (ART) ^5^. However, with current global ART coverage of about 60% ^6^ and over 50% of CM cases occurring in ART-experienced patients ^7^, the incidence of CM remains high ^4^, and timely CM diagnosis and optimal CM treatment are cornerstones for patient survival. Within the last decade, there have been major advances in the diagnosis of CM with the development of highly sensitive and highly specific point-of-care tests capable of providing results within 10 minutes ^8, 9^. However, from a therapeutic perspective, no new medication for the treatment of CM has been developed or approved within the past two decades. Consequently, CM treatment still relies on three off-patent medications, each more than 25 years old: amphotericin B (AmB) and liposomal amphotericin B (L-AmB), flucytosine (5FC) and fluconazole (FLU) ^10, 11^. Clinically, CM presents as a sub-acute infection that requires prolonged treatment divided in three phases: induction, consolidation and maintenance ^11^. The induction phase, which aims at drastically reducing cerebrospinal fluid (CSF) fungal burden within the first 2 weeks, is crucial for good patient outcomes ^12, 13^. As such, the major challenge has been how best to use the limited antifungal treatment arsenal to optimise the induction phase for better patient survival. In low- and middle-income countries where AmB and 5FC are not always readily available, FLU monotherapy even at the highest doses has been associated with over 50% mortality during the first 10 weeks of treatment ^14– 16^, meaning that FLU monotherapy is not a suitable option for induction treatment of CM ^17^. In this review, we summarise the major trials carried out within the last 5 years using combination antifungal therapy for the induction treatment of HIV-associated CM.

## Recent advances in antifungal combination therapies

One of the first randomised controlled trials comparing combination induction therapy for HIV-associated CM using AmB deoxycholate (AmBd) at a higher dose of 1 mg/kg per day instead of the previously used 0.7 mg/kg per day was carried out in Vietnam ^18^. This three-group open-label trial, which enrolled 299 patients, aimed to determine whether 2-week combination therapy with either AmBd plus 5FC (at a dose of 100 mg/kg per day) or AmBd plus FLU (at a dose of 400 mg/day) offered survival benefits over 4-week AmBd monotherapy. Fewer deaths occurred in the AmBd plus 5FC arm at day 14 (hazard ratio [HR] 0.57, 95% confidence interval [CI] 0.30–1.08, *P* = 0.08) and at day 70 (HR 0.61, 95% CI 0.39–0.97, *P* = 0.04). However, AmBd plus FLU did not show any significant survival benefits compared with AmBd monotherapy at days 14 and 70 (HR 0.78, 95% CI 0.44–1.41, *P* = 0.42 and HR 0.71, 95% CI 0.45–1.11, *P* = 0.13 respectively). This trial confirmed 14 days of AmBd 1 mg/kg per day plus 5FC 100 mg/day as standard induction therapy for HIV-associated CM.

Nevertheless, the challenges of delivering 2 weeks of AmBd and AmB-related severe adverse events (SAEs) limit routine use of this regimen in resource-limited settings where monitoring and treating these SAEs are challenging ^19^. Phase II trial results demonstrated that shorter-course AmBd was associated with fewer SAEs than a 2 week course, without diminution in rates of fungal clearance in the second week probably due to long half-life of AmBd in the brain ^20, 21^. Additionally, the oral combination of 5FC and high-dose FLU (1200 mg/day) was shown to have fungal clearance rates approaching those seen with AmBd alone ^22^. These options of short-course combination therapy were tested in an open-label phase 3 randomised non-inferiority multicentre Advancing Cryptococcal meningitis Treatment for Africa (ACTA) trial. ACTA was powered to compare the day 14 and day 70 survival rates of a short course of 1-week of AmB (combined with either high-dose FLU or 5FC in 1:1 ratio) and 2 weeks of oral combination (5FC plus high-dose FLU) with the standard 2-week of AmB combinations ^23^. At 2 weeks, HRs of death were 0.82 (95% CI 0.54–1.25) and 1.01 (95% CI 0.68–1.51) in the oral and 1-week of AmBd groups respectively, compared with the 2-week of AmBd groups. In addition, at 10 weeks, HRs were 0.83 (95% CI 0.61–1.13) for oral and 0.89 (95% CI 0.66–1.21) for the 1-week regimen, in comparison with the standard groups. However, as partner drug to AmB, 5FC was superior to FLU with week-10 mortality (HR 0.62, 95% CI 0.45–0.84, *P* = 0.002). Separate analyses of each regimen as compared with standard 2-week of AmBd plus 5FC showed 1-week of AmBd plus 5FC to have the lowest 10-week mortality (HR 0.59, 95% CI 0.36–0.96) followed by oral combination of FLU and 5FC (HR 0.91, 95% CI 0.63–1.33).

In a recent systematic review and meta-analysis aggregating data from 13 studies encompassing 2426 patients, pairwise analysis showed that, at 10 weeks, 1-week of AmBd plus 5FC is superior to other regimens for the induction treatment of HIV-associated CM and that oral combination of 5FC plus high-dose FLU is the next best option if AmBd is not available or cannot be given safely ^24^.

These two regimens have since been endorsed as the first- and second-line preferred regimens in resource-limited settings in the latest World Health Organization (WHO) guidelines ^11^ ( Table 1).

**Table 1.** World Health Organization 2018 guidelines for the management of cryptococcal disease in adults with HIV.

Induction phase (2 weeks)			Consolidation phase	Maintenance phase (secondary prophylaxis)
Preferred regimen	First week	Second week	Week 3–10	After week 10
	AmB + 5FC	High-dose FLU	Fluconazole (800 mg daily)	Fluconazole (200 mg daily) Until the patient is adherent to ART and antifungal maintenance treatment for at least 1 year and has either - a CD4 count of at least 100 cells/mm ^3^ and a fully suppressed viral load - or a CD4 count of at least 200 cells/mm ^3^
Alternative regimens Depending on availability of drugs	5FC + high-dose FLU			
	AmB + high-dose FLU			
Monitoring and management of raised intracranial pressure are recommended. Routine use of adjunctive corticosteroid therapy is not recommended.			ART initiation should be deferred by 4 to 6 weeks from the initiation of antifungal treatment.	

“High-dose FLU” refers to fluconazole, 1200 mg daily, orally. 5FC, flucytosine (100 mg/kg per day, divided into four doses per day, orally); AmB, amphotericin B deoxycholate (1.0 mg/kg per day, intravenously); ART, antiretroviral therapy. Adapted from World Health Organization. Guidelines for the diagnosis, prevention, and management of cryptococcal disease in HIV-infected adults, adolescents and children. Geneva: World Health Organization; 2018 March 2018 ^11^.

The use of L-AmB is known to be associated with fewer adverse events compared with AmBd formulation ^25^. Again, with the aims of simplifying treatment and reducing adverse events and costs while maintaining efficacy, and based on pre-clinical data showing that L-AmB has a long tissue half-life and effectively penetrates brain tissue ^26^, the Ambisome Therapy Induction Optimisation (AMBITION) phase II trial testing short-course high-dose L-AmB was recently completed ^27^. In Botswana and Tanzania, 80 HIV-infected patients with CM were recruited and randomly assigned to four different arms: the standard 14-day therapy of L-AmB 3 mg/kg per day; a single high dose of L-AmB 10 mg/kg at day 1; two doses of L-AmB: 10 mg/kg at day 1 and 5 mg/kg at day 3; or three doses of L-AmB: 10 mg/kg at day 1 and 5 mg/kg at days 3 and 7. All participants also received high-dose FLU (1200 mg/day). The primary outcome was early fungicidal activity, which was found to be non-inferior to the control arm in all three short-course arms. The overall 10-week mortality was 29% (n = 23), there was no statistical difference between arms, and all arms were well tolerated.

These phase II data have informed the AMBITION phase III trial, which started recruiting in early 2018, using mortality within 10 weeks after randomisation as a clinical primary endpoint and aiming to recruit 850 HIV-infected patients with CM in five SSA countries ^28^. The participants are being randomly assigned to receive either the new WHO-recommended first-line therapy (7 days of AmBd 1 mg/kg per day plus 5FC 100 mg/kg per day followed by 7 days of high-dose FLU 1200 mg/day) or a high single dose of L-AmB 10 mg/kg at day 1, given with 14 days of 5FC and high-dose FLU.

## Recent advances in the management of high intracranial pressure

High baseline intracranial pressure (ICP) of more than 250 mm H _2_O has been shown to be associated with poor short-term survival in patients with CM ^29^. A CSF pro-inflammatory cytokine response with increased levels of tumour necrosis factor-alpha (TNF-α) was suggested to be associated with raised ICP ^30^, and inhibition of pro-inflammatory response with immune modulators like dexamethasone could be associated with a larger reduction in CSF opening pressure during the first 2 weeks of induction therapy ^31^. However, dexamethasone-associated decline in TNF-α levels was also associated with slow fungal clearance and poor outcome, contraindicating the use of dexamethasone in the management of raised ICP ^32^. In a combined cohort on determinants of mortality in 501 patients with HIV-CM, in a context of strict management of high ICP according to guidelines ^30^ with repeated therapeutic lumbar punctures (LPs), high pressures were not associated with increased mortality. In the Cryptococcal Optimal ART Timing (COAT) trial, the effect of therapeutic LP was also found to be associated with improved survival ^33^.

## Recent advances in initiation of antiretroviral therapy

ART initiation is urgently needed in HIV-infected patients with advanced disease ^34^. Even so, paradoxical cryptococcal immune reconstitution inflammatory syndrome still occurs in 15 to 20% of patients with CM after initiating ART ^35^. Generally, cases are diagnosed 1 to 2 months after ART initiation ^36^. Very early initiation of ART (within 3 days of initiation of antifungal therapy) in patients with CM increased mortality compared with a delay of 10 weeks after diagnosis in FLU-treated patients in Zimbabwe ^37^. In the larger COAT trial ^33^, ART-naïve HIV-infected patients with CM were enrolled in Uganda and South Africa and randomly assigned after 7 to 11 days of AmBd plus FLU combination therapy to either early (within 48 hours after randomisation) or delayed (4 weeks after randomisation) initiation of ART. Recruitment was stopped at 177 of the initially planned 500 enrolments because mortality was significantly higher with early initiation (HR 1.73, 95% CI 1.06–2.82, *P* = 0.03); this effect was most marked in patients with low CSF white cell count (HR 3.87, 95% CI 1.41–10.58, *P* = 0.008). A Cochrane systematic review ^38^ including four trials and 294 adults recently concluded that, for HIV-infected patients with CM in low- and middle-income countries, there is a higher risk of all-cause mortality if ART is initiated early (risk ratio 1.42, 95% CI 1.02–1.97).

The management of ART-exposed patients who develop CM remains challenging, and lessons are being learned through ongoing experience. The 2018 WHO guidelines for HIV-associated CM management suggest that initiation, restart or switch of ART be carried out after 4 to 6 weeks of antifungal therapy ^11^.

## Recent advances in adjuvant therapies

Adjuvant steroid therapy is commonly used in HIV-negative patients with pneumococcal or tuberculous meningitis and is associated with improved survival ^39, 40^; however, until recently, the utility of steroids in HIV-associated CM was unknown. In the CryptoDex randomised double-blind placebo-controlled trial ^31^, HIV-infected patients with CM receiving an induction combination therapy of AmB and FLU in six countries in Africa and Asia were randomly assigned to receive either dexamethasone intravenously for the first 2 weeks and then orally until the sixth week or placebo for 6 weeks. Mortality at 10 weeks as the primary outcome showed no difference between the dexamethasone and placebo groups (47% versus 41% respectively; HR 1.11, 95% CI 0.84–1.47, *P* = 0.45). The trial was stopped for safety reasons after the enrolment of 451 out of the planned 880 patients because the data safety monitoring board determined that dexamethasone was causing harm across key outcomes. Dexamethasone was significantly associated with slower rates of decline in CSF fungal count than placebo and was also associated with more adverse events and disability. Recent reports show that this slow fungal clearance and increased mortality could be due to a dexamethasone-induced increased rate of decline in CSF TNF-α ^32^. However, dexamethasone was also significantly associated with a greater reduction in CSF opening pressure during the first 2 weeks than the placebo.

The addition of sertraline, a selective serotonin reuptake inhibitor, to the standard induction therapy has been suggested given limited evidence for *in vitro* activity against *Cryptococcus* ^41^. A phase II dose-finding study ^42^ postulated that adjuvant sertraline therapy might increase cryptococcal CSF clearance compared with historical control data. This was recently tested in the Adjunctive Sertraline for the Treatment of HIV-Associated Cryptococcal Meningitis (ASTRO-CM) randomised placebo-controlled trial in Uganda, where 460 HIV-infected patients with CM receiving combination therapy of AmB and FLU were randomly assigned to receive either sertraline (400 mg/day for 2 weeks and then 200 mg/day for 10 weeks) or placebo in addition to the standard antifungal therapy. The trial was prematurely stopped for futility because 18-week mortality was similar in the sertraline and placebo groups (52% and 46% respectively, HR 1.21, *P* = 0.15) ^43^.

## Conclusions

The management of HIV-associated CM has significantly changed in the past few years. The advent of highly sensitive and highly specific point-of-care tests has reduced diagnostic turnaround time to about 10 minutes. One-week of AmB plus 5FC and 2-week oral combination of high-dose FLU and 5FC are currently preferred first- and second-line induction therapy in resource-limited settings. Simpler induction regimens with L-AmB are being tested in a phase 3 trial. ART initiation following induction therapy should be deferred by 4 to 6 weeks, although managing ART-experienced patients remains a challenge. Dexamethasone or sertraline as adjunctive induction therapy is not recommended. Repeated therapeutic LP is recommended for the management of raised ICP. Progress is being made in access to essential drugs as preferential pricing for L-AmB has been extended from visceral leishmaniasis to CM and wide access to generic 5FC is expected given ongoing personal and international efforts.
